# Defending eugenics: From cryptic choice to conscious selection

**DOI:** 10.1007/s40592-018-0081-2

**Published:** 2018-05-26

**Authors:** Jonathan Anomaly

**Affiliations:** Duke University, Durham, NC USA

**Keywords:** Eugenics, Embryo Selection, Genetic Engineering, Liberal Eugenics

## Abstract

For most of human history children have been a byproduct of sex rather than a conscious choice by parents to create people with traits that they care about. As our understanding of genetics advances along with our ability to control reproduction and manipulate genes, prospective parents have stronger moral reasons to consider how their choices are likely to affect their children, and how their children are likely to affect other people. With the advent of cheap and effective contraception, and the emergence of new technologies for in vitro fertilization, embryo selection, and genetic engineering, it is becoming increasingly difficult to justify rolling the genetic dice by having children without thinking about the traits they will have. It is time to face up to the awesome responsibilities that accompany our reproductive choices.

## Introduction

The title of this essay is deliberately provocative. Eugenics can be thought of as any attempt to harness the power of reproduction to produce people with traits that enable them to thrive. Nearly everyone agrees that parents should provide an *environment* that promotes the welfare of their children. Advocates of eugenics add that we should also manipulate *biology* to promote well-being, provided we can do so without imposing undue risk on our children or on other people with whom they will share the planet.

In defending eugenics, I want to reclaim the spirit of authors like Francis Galton and Charles Darwin, who believed that our reproductive obligations change with our understanding of biology and our capacity to control it. Defending eugenics does not commit us to endorsing state-sponsored coercion, nor to the parochial views held by some advocates of eugenics in the early twentieth century. Likewise, defending eugenics does not commit us to genetic determinism, according to which genes determine every important aspect of our personality. No credible scientist believes this (Sesardic 2005). Rather, the scientific consensus is that virtually every trait that influences our personality and our likelihood of living a good life—including intelligence, health, empathy, and impulse control—has a substantial genetic component (Bouchard 2004; Polderman et al. 2015; Plomin et al. 2016).

I’ll begin with an overview of the problem that motivates eugenics, then describe the widely shared moral principles to which eugenicists have appealed. I’ll end with tentative policy proposals that aim to reverse current dysgenic trends, and increase the extent to which our reproductive choices produce future people who thrive.

## Demographic trends

Reproductive choices constitute a massive intergenerational collective action problem.^1^ In nearly every developed country in the world people who are well-suited to have children have relatively low birth rates, yet future people would be better off if people with heritable traits that we value had a greater proportion of children. The collective action problem that reproductive choices create is much harder to solve than anthropogenic climate change, antibiotic resistance, and other problems with a similar structure. It is also much more dangerous to *try* to solve. Charles Darwin recognized the problem of dysgenic reproductive trends and the perils of possible solutions.^2^ His cousin Francis Galton, a polymath who founded the eugenics movement, shared Darwin’s diagnosis but was more optimistic about solutions.^3^

Darwin argued that social welfare programs for the poor and sick are a natural expression of our sympathy, but also a danger to future populations if they encourage people with serious congenital diseases and heritable traits like low levels of impulse control, intelligence, or empathy to reproduce at higher rates than other people in the population.^4^ Darwin feared that in developed nations “the reckless, degraded, and often vicious members of society, tend to increase at a quicker rate than the provident and generally virtuous members” (Darwin 1882, p. 138).^5^

While Darwin’s language is shocking to contemporary readers, we should take him seriously. The eugenics programs implemented in Nazi Germany are probably the main reason most people no longer acknowledge that there might be some truth to Darwin’s worries. Indeed, because of the racist direction the eugenics movement took in the United States and Germany, many academics after World War II began to deny that races exist, that genes matter, and that intelligence or impulse control are heritable traits that help predict the relative success of different people or groups (Pinker 2002; Cofnas 2016).

As Steven Pinker argues in the context of individual and group differences in intelligence, “in recent decades, the standard response to claims of genetic differences has been to deny the existence of intelligence, to deny the existence of races and other genetic groupings, and to subject proponents to vilification, censorship, and at times physical intimidation” (2006). These are understandable over-reactions to the morally abhorrent policies and pseudo-scientific claims—often couched in the language of eugenics—that led to the Holocaust.

It is striking that in addition to being racist and cruel, Nazi policies had *dysgenic* effects. Hitler’s attempt to exterminate Ashkenazi Jews—arguably among the most intelligent and productive people of the twentieth century^6^—was not only morally outrageous, but contrary to what any reasonable eugenics program would hope to achieve: to produce future people with qualities that we value, including intelligence and creativity. A truly eugenic program might have encouraged Jews to breed more, not less. Hitler’s own vision seems to come from the scientifically erroneous view that there is a “struggle for existence” between races (a frequently misused phrase borrowed from Darwin), and that by virtue of their relative success, European Jews threatened the existence or prosperity of Germans. Hitler’s rise to power came not because of his scientific acumen, but in part because of his ability to scapegoat an especially successful group, and because of the tragic human tendency to commit the zero-sum fallacy (according to which, if one group has more wealth or success, they must have taken it from other groups, rather than adding to the stock of social value).^7^ We should continue to learn from this episode in history, but stop allowing it to silence any discussion of the merits of eugenic thinking.

In fact, there is increasingly good evidence that Darwin was right to worry about demographic trends in developed countries. The evidence is sparse because many people who pursue this research have a hard time getting it funded or published, due to common worries that it will resurrect racism, classism, and intolerant forms of eugenics. But evidence exists. For example, a number of authors have found a negative correlation between IQ and fertility,^8^ between education and fertility, and, independently, between income and fertility—especially in developed countries with robust welfare states and increased opportunities for ambitious and intelligent women.^9^ The problem is exacerbated by the fact that people with more education and income (correlated with higher intelligence), tend not only to have fewer children but also tend to delay reproduction in the pursuit of other goals.

This is consistent with Hermann Muller’s observation that “it is not the having of children but the prevention of them which today requires the more active, responsible effort, an effort which makes demands on the participants’ prudence, initiative, skill, and conscience” (1963, p. 253). By contrast, Muller maintains, “persons possessed of greater foresight, and those with keener regard for their family, usually aim to have a lower than average number of children, in order that they may obtain higher benefits for those children that they do have, as well as for themselves and those near to them.” (1963, p. 254).

The demographic pattern Muller identified is partly explained by the high opportunity cost of having children in societies in which successful parents can pursue other goals (Becker 1981). Another explanation is that when especially successful people have fewer children, other ambitious people emulate them (Richerson and Boyd 2005, ch. 5). For example, it is increasingly common for ambitious and compassionate career women to choose to have pets rather than children, or to adopt children in middle age rather than having their own. This trend may have good effects on the adopted children in the short run but bad effects on the gene pool over the long run. Substituting cats for kids, or devoting one’s career to caring for other people’s kids while foregoing reproduction oneself, may be an example of pathological altruism (Oakley 2012). This occurs when pro-social emotions that were adaptive in ancestral environments produce behavior that is either counter-adaptive or leads to its own long-run reduction in modern environments.

Whatever the evidence for dysgenic trends in developed countries, Francis Galton tried to show in his book *Hereditary Genius* (1869) that qualities we care about tend to run in families, and that changing the norms surrounding reproduction could dramatically improve the human population in the same way artificial selection can improve domesticated animals.^10^ Galton’s followers included playwright George Bernard Shaw, novelist HG Wells, and the evolutionary biologist JBS Haldane.^11^ At the turn of the twentieth century, an increasing number of influential intellectuals sought to promote education about heredity and shape social norms so that women would be encouraged to carefully choose the fathers of their children. Some of the more fervent eugenicists, many of whom overestimated their understanding of the relevant science, began to promote statutes that would allow states to involuntarily sterilize citizens deemed unfit for reproduction. The first eugenic sterilization law was passed in Indiana in 1907. By the time Virginia passed a similar law in 1924, it was following the lead of 15 other American states.

## Moral Principles

In 1927 the United States Supreme Court voted by an 8-1 margin to uphold the state of Virginia’s right to sterilize “feeble-minded” citizens. While the language of *Buck v Bell* may seem callous, and the evidence in the case was flimsy, the moral foundations of the decision are defensible. Writing for the majority, Justice Holmes argued that the Virginia statute was premised on the ideas that “the health of the patient and the welfare of society may be promoted in certain cases by the sterilization of mental defectives…without serious pain or substantial danger to life…”^12^ Controversial as this statement is, on a charitable reading, the moral principles the court highlights include coercing “mental defectives” (who can’t make competent choices) to undergo surgery only if it involves little danger or pain, and if it either makes the person being coerced better off, or prevents them from bearing children who are likely to impose significant harm on future people.

In the penultimate paragraph, Holmes compares the sacrifice of someone who is involuntarily sterilized with the sacrifice of soldiers drafted into war:

We have seen more than once that the public welfare may call upon the best citizens for their lives. It would be strange if it could not call upon those who already sap the strength of the State for these lesser sacrifices, often not felt to be such by those concerned, in order to prevent our being swamped with incompetence. It is better for all the world if, instead of waiting to execute degenerate offspring for crime or to let them starve for their imbecility, society can prevent those who are manifestly unfit from continuing their kind. The principle that sustains compulsory vaccination is broad enough to cover cutting the Fallopian tubes.^11^

In the final line of the decision Justice Holmes cites an earlier case (*Jacobson v. Massachusetts*) in which the Supreme Court upheld a law that required a Swedish immigrant to vaccinate his children against smallpox (despite the father’s objections) in order to prevent serious harm to the child, and through the child, other people.

While the language of the decision is compatible with a variety of moral theories, the core principle is that a citizen can be required to undergo a procedure if the cost to him is trivial compared to the social benefits. Nearly *all* moral theories hold this view, though people disagree about the magnitude and certainty of benefits we would need in order to justify the expected costs to the person being vaccinated, sterilized, or otherwise coerced. Compulsory sterilization is far more controversial than compulsory vaccination because of the degree of invasiveness, and the potential for abuse. Nevertheless, the following moral principles seem to be expressed in *Buck v Bell*:

1. The state may (in some cases) restrict someone’s liberty if their mental capacity undermines their ability to make voluntary choices, and if their choices put them at serious risk of causing far-reaching and irreversible *harm to themselves*.
2. The state may (in some cases) restrict someone’s liberty when leaving them free to act as they wish poses serious risks of *harm to others*.
3. The state may (in some cases) require us to act in ways that promote social welfare when we find ourselves in collective action problems in which each of us has an incentive to act one way, but most of us are better off if most people act in another way.
4. When the state has compelling reasons to coerce its citizens in accordance with one or more of the three principles above, it should do so in a way that restricts liberty least, and involves the least amount of pain or sacrifice.

The plausibility of these principles in other contexts strongly suggests that much of the vehement rejection of eugenic policies after the Second World War was about empirical assumptions rather than moral principles. More specifically, people disagree about issues like the extent of our knowledge of genetics, the safety of eugenic procedures, and the ability of government agents to make the right call on whether a particular person or group has heritable characteristics that are likely to be transmitted to children who will live very bad lives, or adversely affect other people. I argue that we can use these moral principles to inform a more cautious approach to eugenics which places more weight on individual liberty and less confidence in the wisdom of state agents than early manifestations of eugenics did.^13^ Some call this approach *liberal* eugenics (Agar 2004).

## Policy proposals

Many people distinguish negative from positive eugenics, and coercive from non-coercive eugenics. The idea is that negative eugenics tries to sift out undesirable psychological or physical characteristics (like psychopathy or Tay Sachs disease), while positive eugenics seeks to increase the prevalence of traits that promote individual and social welfare (like creativity or a healthy immune system).^14^ Coercive eugenics uses force to achieve these ends, while non-coercive eugenics uses education, information, and social norms to achieve them. The distinctions are not sharp, and they do not map onto what is right or wrong in any obvious way (Gyngell and Selgelid 2016). It is best, then, to focus on the justifiability of particular public policy proposals.

### Free contraception

The advent of reliable contraception—colloquially called “birth control”—was seen by some of its greatest proponents as a way to liberate women: to give them control over their lives by freeing them from the shackles of continual pregnancy and the consequences of rape. More generally, contraception allows women to invest in education rather than cosmetics, which is good for them, and for society. But some of birth control’s most famous proponents also saw the potential for it to have eugenic effects, since it allows women to decide who fathers their children, and when to have children. Contraception, in other words, allows women to have sex for fun, but to carefully decide when and how and with whom to have children.

According to Margaret Sanger, founder of Planned Parenthood, “Birth Control…means not merely the limitation of births, but the application of intelligent guidance over the reproductive power. It means the substitution of reason and intelligence for the blind play of instinct” (1922, ch. 2). Without contraception, Sanger feared, civilization “will be faced with the ever-increasing problem of feeble-mindedness, that fertile parent of degeneracy, crime, and pauperism” (1922, ch. 4). While Sanger’s language is harsh, her point is plausible.^15^ Contraception can prevent unwanted pregnancies, the social consequences of which are borne broadly.^16^ Since each of us has an interest in promoting an environment in which current and future people flourish, there are good reasons to make contraception freely available for all. This is one of the most cost-effective measures governments can take, and it can be justified by its ability to enhance individual autonomy and social welfare.

### Genetic education and counseling

The division of cognitive labor that market society occasions allows us to accumulate vast amounts of knowledge, but it also renders people (rationally) ignorant about how most things work, including the universe in general, and the human body in particular (Hayek 1945). Most people apparently do not *want* to understand cosmology, biology, or genetics. And they don’t need to in order to live reasonably successful lives, even if their success depends on other people knowing little bits about how things around them work—like microwave ovens, human kidneys, nuclear power plants, internal combustion engines, etc. All of us depend on these things working well, and on the ability of experts to repair or replace them when they fail, but very few people need to know how any or all of these work.

The problem comes when we bear the costs of other people’s ignorance. In a democracy we share the undesirable policy consequences of one another’s ignorant or irrational votes (Huemer 2015). And in a society that shares at least some of the costs and benefits of productive work, the consequences of people reproducing at random are felt by all of us. This suggests that taxpayers should be willing to finance genetic testing and the provision of genetic information to prospective parents, with the goal of helping them make informed reproductive choices that will benefit their children, and protect other people from harm. Since state provision of education or information always has the potential to turn into propaganda (Mill 1859, ch. 5), there may be reasons to publicly finance its *private* provision by teachers and doctors in a competitive market. Finally, in addition to education, as genetic engineering becomes safe and affordable, barriers to accessing socially beneficial genetic enhancements should be removed.

### Incentives and penalties

The current demographics of Western countries are troubling, as people with a higher IQ, more education, and greater income reproduce at relatively low levels. Some have suggested paying people who would make good parents to have children. But this misunderstands the problem of opportunity cost: most people with higher intelligence or more money don’t reproduce less because they can’t afford children; they do so because they have many other valuable ways of spending their time, including writing books, volunteering, taking exotic vacations, and advancing their careers.

States might improve the situation by mandating paid parental leave in the workplace, so there are fewer costs to temporarily leaving work to take care of children. Sweden has among the most generous paid parental leave laws in the world, and it is among the few developed countries with a replacement birthrate. Some studies suggest that strong family leave laws are the primary reason for its demographic stability (Hoem 2005). But the evidence is tainted by the fact that native-born Swedes have below-replacement fertility, and foreign-born immigrants from Somalia have more children, thus bringing Sweden close to replacement levels (Tollebrant 2017). This makes the effect of family leave laws a little unclear.

Some authors have suggested paying some people *not* to reproduce, or instituting a parental licensing scheme. Francis Crick tentatively proposed both ideas at a symposium on eugenics (1963, pp. 276, 284).^17^ In principle, there are reasons to support policies like these. But they raise real worries about corruption by bureaucrats, black markets for pregnancy, and political legitimacy: in constitutional democracies, controversial policies cannot produce their desired effects over the long run unless there is some degree of transparency and public support.

The rationale for any justified licensing scheme is that some activities require competence to safely perform, and those who engage in them without adequate skill or foresight are likely to seriously harm other people (LaFollette 1980). Some parents lack the desire to take care of their children. This is illustrated by the fact that many single mothers sue unwilling fathers for court-mandated child support. Other parents abuse their children, or lack the means to provide food, shelter, medical care, and education to their children.

The typical response is for the state to step in and pay for all of these things, and in extreme cases to remove children from their parents and put them in foster care. But it would be more cost-effective to *prevent* unwanted pregnancies than treating their consequences, especially if we could achieve this goal by subsidizing the voluntary use of contraception. It may also be more desirable from the standpoint of future people.

The most compelling reason (though certainly not a *decisive* reason) for supporting parental licensing is that traits like impulse control, health, intelligence, and empathy have significant genetic components.^18^ What matters is not just that some parents are unwilling or unable to take care of their children; but that in many cases they are passing along an undesirable genetic endowment.

As John Stuart Mill argued:

It is not in the matter of education only, that misplaced notions of liberty prevent moral obligations on the part of parents from being recognised, and legal obligations from being imposed… The fact itself, of causing the existence of a human being, is one of the most responsible actions in the range of human life. To undertake this responsibility—to bestow a life which may be either a curse or a blessing—unless the being on whom it is to be bestowed will have at least the ordinary chances of a desirable existence, is a crime against that being (1859, ch. 5).

Mill expressed ambivalence about imposing coercive laws on parents, but was clear that policies requiring prospective parents to demonstrate their ability to care for children “are interferences of the State to prohibit a mischievous act—an act injurious to others, which ought to be a subject of reprobation, and social stigma, even when it is not deemed expedient to superadd legal punishment” (1859, ch. 5).

For a parental licensing scheme to be fair, we would need to devise criteria that are effective at screening out only parents who impose significant risks of harm on their children or (through their children) on other people. This is hard enough. It would be even more difficult to select appropriate penalties to impose on those who fail a reproductive licensing test, but have children anyway. One way to enforce licensing is to impose fines or other costs on those who have children without a license. Despite its unpopularity in many states around the world, American judges occasionally order “deadbeat dads” who father children they can’t support to stop reproducing, though the order is difficult to enforce, especially since most states lack eugenic sterilization provisions. As a rule of thumb, though, states should apply as little coercion as possible to facilitate the emergence of future people with traits that enable them to thrive.^19^

## Conclusion

Public policies cannot create a eugenic utopia. In fact, passing legal mandates and licensing requirements is often more dangerous and less effective than relying on voluntary choice to achieve the same results. It may be desirable to increase informed consumer choice by subsidizing contraception and improving access to education about genetics and reproductive technology so that people can make conscious choices about the characteristics of their children. Changing reproductive norms can also go some way in encouraging eugenic choices. For example, as it becomes more socially acceptable to use sperm and egg donors, to screen embryos, and to use surrogates, the outcome will likely be collectively beneficial. Many people who have a visceral fear of these procedures are even more apprehensive about genetically modifying embryos. But arguments can change attitudes.

In recent years, influential authors have argued that we have a moral obligation to produce children with the best chance of the best life (Savulescu and Kahane 2009), and that many opponents of biomedical technology have a discredited teleological view of human evolution (Buchanan and Powell 2011). I have offered a guardedly optimistic account of how some public policies might increase the extent to which our reproductive choices are both individually rational and collectively desirable. But I concede that I may be wrong about any of the measures I’ve considered. Sometimes the best policy is not to have one.

## Footnotes

1 I elaborate on this claim in an earlier essay (Anomaly 2014).

2 By “dysgenic” I mean the proliferation of people with traits that are detrimental to human welfare. An example would be a trait like sadism, psychopathy, or extremely low intelligence.

3 Though Galton’s project was not new. In the *Republic*, Socrates asks Glaucon “if care was not taken in their breeding, your dogs and birds would greatly deteriorate, right?” Glaucon agrees, and Socrates continues, “the best men must have intercourse with the best women as frequently as possible, and the opposite is true of the very inferior.” *Republic*, 459e, first published around 380 BCE.

4 A similar worry that Darwin could not have understood is the problem of “mutational meltdown” first explored by Hermann Muller, and discussed by evolutionary biologist John Tooby (2016). The problem is that without the brutal checks of natural selection, mutation load tends to increase across generations.

5 Darwin expresses some uncertainty whether this is true in contemporary Britain, but concludes that “If the various checks [of infectious disease, starvation, and involuntary celibacy] do not prevent the reckless, the vicious and otherwise inferior members of society from increasing at a quicker rate than the better class of men, the nation will retrograde, as has occurred too often in the history of the world. We must remember that progress is no invariable rule” (1882, p. 140).

6 Informal evidence for this claim comes from the success of Jews around the world even in the presence of social and legal discrimination, and from the percentage of Nobel prizes and other scientific accolades Jews were awarded in the twentieth century. More rigorous evidence comes from the heritability of IQ scores (Ashkenazi IQ is the highest in the world, nearly two standard deviations above the global average). For more on the evolution of Ashkenazi intelligence, See Cochran et al. (2006), Cochran and Harpending (2009, ch. 7), and Wade (2014, ch. 8).

7 As Nicholas Wade points out, “Like Chinese immigrant communities, Jews have brought enormous benefits to the economies in which they worked. Unfortunately their success, like that of the immigrant Chinese, has in many cases elicited not gratitude but envy, followed by discrimination or murderous reprisals, a response that reflects more strongly on the greed than the intelligence of their host populations” (2014, p. 213).

8 Demographers use “fertility” to refer to the number of children people choose to have, not their capacity to have children.

9 See for example Teasdale and Owen (2008), Meisenberg (2009, 2010), and Lynn and Harvey (2008). Intelligence researchers acknowledge the so-called Flynn effect, which refers to the rise in IQ throughout the world due to environmental improvements like clean water and nutrition, but argue that in developed countries these are already exhausted or near exhaustion, so that the genetic component of IQ may be sinking even as the environmental component has risen. I should emphasize that IQ is an imperfect measure of intelligence, and that intelligence is not the only thing that matters. An intelligent sadist makes him more dangerous rather than more admirable.

10 Showing that a trait runs in families was an imperfect way of showing a genetic component in an age before genomics. This method can still yield useful results. For example, economist Gregory Clark (2014) uses more sophisticated data analysis than Galton had available to try to disentangle social and genetic factors in explaining social mobility and success.

11 A character in Bernard Shaw’s play *Man and Superman* lamented that “The day is coming when the great nations will find their numbers dwindling from census to census; when the six-roomed villa will rise in price above the family mansion; when the viciously reckless poor and the stupidly pious rich will delay the extinction of the race only by degrading it; while the boldly prudent, the thriftily selfish and ambitious, the imaginative and poetic, the lovers of money and solid comfort, the worshippers of success, of art, and of love, will all oppose to the Force of Life the device of sterility” (1903, p. 159).

12 *Buck v Bell*, 274 US 200 (1927).

13 Versions of these principles can be found in recent work on the ethics of reproduction. See Dan Brock (2005), and David Benatar (2011). Many critics of eugenics have been so concerned with condemning past policies, that they often fail to distinguish moral principles from empirical claims (Buchanan 2007). This has led to moral grandstanding and motivated reasoning among its critics, leading many to minimize the importance of genes in shaping personality traits and capacities.

14 Whether we describe an action as “promoting social welfare” or “not reducing social welfare” depends on where we draw the baseline. The baseline problem suggests that we cannot come up with a simple answer in distinguishing “making better” and “not making worse.” In thinking about whether we are promoting welfare or preventing harm it is always worth asking, “compared to what?”.

15 Sanger’s claim about the link between genes and crime, long ignored or repudiated by academics, appears to be borne out to some extent by twin studies. See Barnes et al. (2014).

16 The social consequences of reproductive choices spill across borders and generations. This suggests that citizens in wealthy countries have strong reasons to support subsidizing contraception for those in poor countries. In particular, the population in most African countries is predicted to explode over the next century, and while population growth per se is not a bad thing, it is bad when it occurs in countries that rely on Western technology for food and medicine, but that cannot support their own population through endogenous economic growth within stable political institutions.

17 The conference was attended by other Nobel laureates like Hermann Muller and JBS Haldane, and public intellectuals like Julian Huxley whose brother Aldous wrote the eugenic dystopia *Brave New World*.

18 Scientists distinguish the *cognitive* and *affective* components of empathy (the first concerns our ability to understand other people, especially their emotions, and the second concerns our ability to react appropriately to someone else’s emotional states). Both are strongly influenced by genes, and the absence of either or both is associated with narcissism, psychopathy, or autism (Baron-Cohen 2012). On the other hand, too much empathy can lead to neuroticism and pathological altruism. So there is presumably an optimal range of empathy outside of which children are more likely to develop disorders.

19 To the extent that informal social sanctions work, these are preferable to coercive laws. But informal sanctions often fail to work well in large and anonymous groups, especially when the costs and benefits of our choices are spread thinly, and accrue mainly to future people. This may give us reason to endorse more freedom to move between political communities with different rules and norms (Nozick 1974, ch. 10). But this freedom wouldn’t address the demographic worry that the proportion of people in the world making eugenic reproductive choices may be shrinking, which is a problem for distant generations of people who share a single planet, if not a single political community.
